# Asthma Inception: Epidemiologic Risk Factors and Natural History Across the Life Course

**DOI:** 10.1164/rccm.202312-2249SO

**Published:** 2024-07-09

**Authors:** Erik Melén, Heather J. Zar, Valerie Siroux, Dominic Shaw, Sejal Saglani, Gerard H. Koppelman, Tina Hartert, James E. Gern, Benjamin Gaston, Andrew Bush, Joe Zein

**Affiliations:** ^1^ Department of Clinical Science and Education, Södersjukhuset, Karolinska Institutet, Stockholm, Sweden;; ^2^ Department of Paediatrics and Child Health and South African Medical Research Council Unit on Child and Adolescent Health, University of Cape Town, Cape Town, South Africa;; ^3^ University Grenoble Alpes, Inserm U 1209, CNRS UMR 5309, Team of Environmental Epidemiology Applied to Development and Respiratory Health, Institute for Advanced Biosciences, Grenoble, France;; ^4^ Department of Respiratory Sciences, University of Leicester, Leicester, United Kingdom;; ^5^ National Heart and Lung Institute, Centre for Paediatrics and Child Health, Imperial College London, London, United Kingdom;; ^6^ Department of Pediatric Pulmonology and Pediatric Allergology, Groningen Research Institute for Asthma and COPD, University of Groningen, University Medical Center Groningen, Beatrix Children’s Hospital, Groningen, the Netherlands;; ^7^ Department of Medicine and Pediatrics, Vanderbilt University School of Medicine, Nashville, Tennessee;; ^8^ Department of Pediatrics, University of Wisconsin, Madison, Wisconsin;; ^9^ Indiana University, Indianapolis; and; ^10^ Mayo Clinic, Phoenix, Arizona

**Keywords:** allergy, child, genetics, epidemiology, risk factor

## Abstract

Asthma is a descriptive label for an obstructive inflammatory disease in the lower airways manifesting with symptoms including breathlessness, cough, difficulty in breathing, and wheezing. From a clinician’s point of view, asthma symptoms can commence at any age, although most patients with asthma—regardless of their age of onset—seem to have had some form of airway problems during childhood. Asthma inception and related pathophysiologic processes are therefore very likely to occur early in life, further evidenced by recent lung physiologic and mechanistic research. Herein, we present state-of-the-art updates on the role of genetics and epigenetics, early viral and bacterial infections, immune response, and pathophysiology, as well as lifestyle and environmental exposures, in asthma across the life course. We conclude that early environmental insults in genetically vulnerable individuals inducing abnormal, pre-asthmatic airway responses are key events in asthma inception, and we highlight disease heterogeneity across ages and the potential shortsightedness of treating all patients with asthma using the same treatments. Although there are no interventions that, at present, can modify long-term outcomes, a precision-medicine approach should be implemented to optimize treatment and tailor follow-up for all patients with asthma.

**Contents**

From Wheeze to Asthma
  Early Wheezing and the Role of Viral/Bacterial Infections
  Early-Life Asthma Pathophysiology and Disease Persistence
  Family History of Asthma and the Contribution of Genomics
Risk Factors
  Perinatal and Early-Life Postnatal Exposures
  Lifestyle Factors, Environmental Exposures, and Climate Change
  Medications
Asthma Across the Life Course
  Epidemiology of Asthma during the Life Course
  Adolescent- and Adult-Onset Asthma
  Exercise-induced Bronchoconstriction and Asthma
Prevention of Asthma Onset and Progression of Established Disease
  Primary Prevention of Asthma
  Transition from Childhood to Adulthood
  The Evolution of Severe Asthma across the Life Course
  Biologics as Disease-Modifying Drugs
Conclusions

“Asthma” is a descriptive label for a lower airway disease manifesting with symptoms including breathlessness, cough, difficulty in breathing, and wheezing, which is characterized by combinations of acute attacks and interval symptoms (1). Asthma is characterized by any or all of varying types of airway inflammation, airway hyperresponsiveness, mucus hypersecretion, and an abnormal airway response to pathogens, all leading to variable airflow obstruction. Asthma is an umbrella term, and it is likely that different pathophysiological mechanisms lead to the clinical presentation and diagnosis. When feasible, age-appropriate lung function tests should be used to demonstrate variable airflow obstruction (and confirm asthma diagnosis from age 5 yr), and biomarkers and other investigations should be done to define clinical (phenotype) and biological (endotype) disease characteristics (i.e., the type of asthma) (2). Heredity remains a very important risk factor for disease development (3), in addition to early-life (i.e., from conception to first 3 yr of life postnatally) conditions, such as recurrent airway infections, wheezing, and multiple aeroallergen IgE sensitization (4). Several lifestyle and environmental risk factors for asthma have been identified, including maternal/parental tobacco smoke exposure, air pollution, allergens, social deprivation, and, more recently, additional risk factors including chemical pollutants (e.g., phenols, phthalates) and extreme weather events related to climate change (5).

The asthmas are truly complex diseases, and, like many chronic diseases, there is no single determinant or causal factor. Despite major progress in experimental and clinical research, there are still many knowledge gaps in asthma inception and progression across the life course. What is the childhood asthma prevalence as of today, and when does asthma commence? Is there an early-life disease state before the onset of airway inflammation? What are the key drivers from childhood wheezing to asthma? Can we identify reliable biomarkers that enable distinction of different asthma phenotypes (beyond fractional exhaled nitric oxide [FeNO] and blood eosinophils)? What is the long-term prognosis given the patient’s type of asthma, and can we prevent asthma from becoming severe? In this review, we address some of these issues, with a focus on asthma inception and progression across the life course, from mechanisms to epidemiology and clinical applications. We have not covered asthma remission, which will be the subject of another review.

## From Wheeze to Asthma

### Early Wheezing and the Role of Viral/Bacterial Infections

Acute wheezing in early life is most often caused by viral infections, such as respiratory syncytial virus (RSV), rhinovirus (RV), and metapneumovirus, and can be the first manifestation of childhood asthma (6). RSV is the most likely culprit in the first year or two (6), and personal risk factors for more severe illnesses include preterm birth, season of birth, genetic predisposition, and premorbid reduced lung function (1). Two prospective observational studies of prophylactic treatment of preterm infants with palivizumab, an RSV monoclonal antibody licensed to prevent severe RSV lower respiratory tract infection (LRTI) in select high-risk infants, have demonstrated similar and significantly lower odds of physician-diagnosed recurrent wheeze in the first 6 years of life (adjusted odds ratio [aOR], 0.34; 95% confidence interval [CI], 0.19 to 0.60; and aOR, 0.31; 95% CI, 0.12 to 0.81) (7, 8). In the first study, palivizumab significantly reduced recurrent wheezing in children with a family history of allergy (aOR, 0.48; 95% CI, 0.26 to 0.90) but not in those with no family history of allergy (aOR, 0.55; 95% CI, 0.20 to 1.55). In contrast, in the second study, receipt of RSV prophylaxis was associated with a significant reduction in recurrent wheezing among children with no family history of atopy (aOR, 0.31; 95% CI, 0.12 to 0.81) but did not have a significant effect in infants with a family history of atopy (aOR, 0.69; 95% CI, 0.34 to 1.39), suggesting that RSV predisposes to recurrent wheezing through atopy-independent mechanisms. In a small underpowered study (but the only randomized trial of RSV prophylaxis that, to date, has followed children to the outcome of asthma), among 429 infants, a protective effect was not observed on doctor-diagnosed asthma (absolute risk reduction of −0.4; 95% CI, −6.5 to 5.8) or lung function at age 6 years—only on wheeze, parent-reported asthma, and asthma medication use (9, 10). However, RSV prophylaxis does not prevent infection, only severe infection, and the association of severe RSV infection and asthma is likely confounded by shared heredity (11). This is important, because regardless of infection severity, only one study has examined the effect of absence of RSV infection on asthma risk. Not having RSV infection in the first year (by surveillance or serology) has been associated with a 26% reduced risk (adjusted relative risk, 0.74; 95% CI, 0.58 to 0.94) of developing asthma by age 5 years, with the greatest risk reduction for nonatopic asthma (12).

RV can also cause bronchiolitis and is the most frequent cause of wheezing illnesses in preschoolers and older children (6). Risk factors for RV wheezing in preschool children include low lung function and atopic characteristics, such as eczema and allergic sensitization (13). RV illnesses cause airway inflammatory and metabolic responses that are distinct from RSV (14, 15), which may have clinical implications. For example, current guidelines recommend against the use of corticosteroids in bronchiolitis because of lack of efficacy (16). However, there is limited evidence that prednisolone hastens improvement in children with severe bronchiolitis caused by RV, but not other viruses (17, 18). RV wheezing is associated with the highest risk for subsequent asthma development (6), especially in atopic children (19). In the absence of preventive treatments, whether RV causes or reveals asthma is uncertain. However, there are clues that this relationship may be causal. For example, a polymorphism in *CDHR3*, which encodes the RV-C receptor, increases its expression on the cell surface and confers increased susceptibility to RV-C infection and wheezing illnesses (20–22). This polymorphism is also a risk factor for a phenotype of childhood asthma with recurrent wheezing episodes (23).

Both viral and bacterial pathogens have similar associations with wheezing illnesses in childhood. Nasal microbiota dominated by some normal colonizing bacteria that can act as opportunistic respiratory pathogens (e.g., *Moraxella catarrhalis*, *Haemophilus influenzae*, *Streptococcus pneumoniae*, and *Staphylococcus aureus*) may increase the risk of preschool wheeze and subsequent asthma (24–26). Viral illnesses can lead to an increased abundance of these pathogens, which is then associated with a greater probability of illness, including wheezing (24, 25, 27). Conversely, upper airway microbiota dominated by commensal bacteria such as *Corynebacterium* and *Dolosigranulum* are associated with a lower risk of subsequent wheezing and asthma (24, 25). Analysis of temporal patterns of microbial colonization in the first 6–12 months suggests that the sequence of microbiome development is important (24, 28) and that antibiotic therapy can disturb this progression and promote pathogens (*see* below about medications) (29, 30).

Viral and bacterial wheezing illnesses interact with mucosal immune responses. Allergic inflammation can suppress antiviral responses, leading to increased viral illnesses and wheezing LRTI (31, 32). Viral infections and airway bacteria can also stimulate local immunity (33, 34). Bacterial stimulation of mucosal immune responses may be necessary to help the immune system develop healthy tolerance and antipathogen responses (35). Social distancing, including school closures during the pandemic, led to markedly lower rates of childhood respiratory illnesses, including wheezing. When the coronavirus disease (COVID-19) pandemic restrictions eased, outbreaks of illnesses with common viruses (RV, RSV) exceeded prepandemic levels in some countries (36). These patterns suggest that common respiratory illnesses may prime (or “train”) innate and adaptive antiviral responses to increase their effectiveness (37).

Childhood parasitic infections, more common in lower- and middle-income countries (LMICs), can inhibit proinflammatory immune responses (38). Several studies have associated geohelminth infections (other than *Toxocara*) with reduced wheeze and childhood asthma (39, 40). The timing of infection also relates to clinical outcomes. In a longitudinal study enrolling families just after birth, maternal prenatal geohelminth infections were associated with increased wheeze in offspring. In contrast, children with infections in the first 3 years had reduced wheeze and nonallergic asthma (41).

Fungal pathogens and the mycobiome are increasingly recognized to have important immunomodulatory functions throughout early life, influencing asthma development (42). Residential exposure to mold in early life has been dose-dependently associated with persistent wheezing later in childhood (43), and increased long-term exposure to damp has been associated with development of asthma (44).

### Early-Life Asthma Pathophysiology and Disease Persistence

The failure to recognize disease heterogeneity in childhood asthma has limited progress in treatment approaches, especially for children less than 5 years old. In infancy, the first episode(s) with these symptoms may be labeled bronchiolitis; however, approximately 40% of infants who have had at least one wheezing episode will have recurrent symptoms and attacks through the preschool years, and a further one-third will progress to develop symptoms through to school age. Recurrent attacks of at least two episodes of wheezing put children at risk for future asthma diagnosis; these children should be further characterized to address the type of wheezing. These tests may include IgE sensitization to aeroallergens, blood eosinophils, and a treatment trial of inhaled corticosteroids.

Preschool-aged children with recurrent, severe wheezing have several pathophysiological phenotypes (and associated endotypes). Unbiased analysis of lower airway BAL pathology in 103 children with severe preschool asthma (median age, 3 yr) showed distinct clusters, including an allergic cluster (aeroallergen sensitization, blood eosinophilia, predominance of lower airway neutrophils, and *M. catarrhalis* infection) and a nonallergic cluster (no aeroallergen sensitization, predominance of lower airway neutrophils, and bacterial infection distinct from the allergic cluster, with *S. aureus*, *H. influenzae*, and *S. pneumoniae*) (45). All had a high prevalence of lower airway RV detection. A similar approach assessing BAL inflammation in children ranging from 1 to 16 years showed three clusters: one with a lower airway neutrophilic, steroid-refractory, preschool phenotype; another with severe recurrent preschool wheeze and aeroallergen sensitization; and a third eosinophilic steroid-refractory phenotype with multiple allergic comorbidities (allergic rhinitis, atopic dermatitis, food allergies) at school age (46). Moreover, previous data have shown a subgroup of preschool severe recurrent wheezers have airway wall eosinophilia, which may be independent of atopy (47, 48). Recent evidence suggests that blood neutrophil responses to viral infection differ by allergic phenotype and may be less effective in preschool children without allergic inflammation (49). However, in all these studies, consistency of phenotypes over time is not known.

The key role of multiple exposures, including airway infections (viral, bacterial, and fungal) and allergens, and their impact on balance and function of eosinophils and neutrophils, in determining the pathophysiology and phenotypes of preschool asthma is now becoming apparent. Identification of reliable biomarkers that enable distinction of these infection and inflammatory phenotypes is now an urgent unmet need to enable targeted therapies.

Airway epithelium and smooth muscle play an important role in asthma inception. As originally proposed by Fabry and Fredberg, the biophysical properties of the airway are remarkably similar to those of glass (50). An airway that is repeatedly or chronically contracted becomes “jammed,” or biophysically unable to expand (51). This appears to account for elements of chronic obstruction observed in asthma. Moreover, airway cilia themselves appear to contribute to asthma pathophysiology (52). For example, decreased ciliary motion decreases production of S-nitrosoglutathione and other beneficial nitrogen oxides (53). These factors, in turn, affect both airway inflammation and β_2_-agonist responsiveness (54). Thus, chronic loss of ciliary function—whether genetic or following viral infection—may have a role in asthma pathogenesis.

A hallmark feature of severe, recurrent wheezing and asthma in childhood is the presence of airway remodeling (AR) (structural airway wall abnormalities including increased subepithelial reticular basement membrane [RBM] thickening, increased airway smooth muscle, and goblet cell metaplasia), which relates closely to lung function (55). A novel approach using unbiased analysis has shown the potential utility of AR parameters (from bronchoscopy) in children aged 1–5 years with severe recurrent wheezing as predictors of severe and frequent exacerbations. The latent class with greatest RBM thickness, vessel density, reduced mucus glands, and RBM to smooth muscle distance were most susceptible to future severe frequent attacks (56). The importance of airway epithelial dysfunction in association with remodeling is also apparent in early life (57). Specifically, only EGFR (epithelial growth factor receptor) and SMAD genes, both of which are associated with epithelial dysfunction and AR, were upregulated in children whose viral upper respiratory infections progressed to asthma exacerbations (58). Moreover, targeted anti-eosinophilic treatment with anti-IL5 antibody in children with moderate-to-severe asthma who had an increased EGFR/SMAD-related nasal epithelial/remodeling gene signature resulted in worsening exacerbations (59). These data support findings from previous randomized clinical trials (60–62) that antiinflammatory therapies alone are insufficient to treat all childhood asthma phenotypes, and, given the intimate link of remodeling with lung function, long-term disease-modifying strategies may only succeed if they can prevent or minimize remodeling.

Bronchial hyperresponsiveness (BHR) shortly after birth is also likely an important determinant of later asthma, with an even bigger signal than baseline airflow obstruction (63). Three studies determined BHR shortly after birth (63–65), before any evidence of airway inflammation is present (66). All three studies showed early BHR was associated with asthma and other adverse respiratory outcomes (63, 67, 68). The pathophysiological basis of the reported BHR is unclear, but in a murine study, nicotine-exposed mice had pups with longer airways than normal and BHR at birth, presumably because the greater baseline resistance of the elongated airways led to a greater reduction in airflow for a given airway narrowing. Airway dysanapsis is defined as a normal FEV_1_, a greater than normal FVC, and thus a low FEV_1_/FVC ratio (69) (i.e., undersized airways relative to the volume of their lungs). Likely, the nicotine-exposed pups mentioned above are a murine dysanapsis model. Dysanaptic airway growth can also result from excessive weight gain in the first 2 years of life (70). Children with high body mass index (and obesity) are known to have larger FVC, a hallmark of dysanapsis, and recent data suggest that obese children also are likely to have peripheral airway impairment (71, 72). Obesity is associated with increased concentrations of proinflammatory cytokines such as IL-6 and is characterized by a type 2 (T2)-low inflammation, airway neutrophilia, and poor response to inhaled corticosteroids (73, 74). Asthma outcomes are worse in children with dysanapsis and, interestingly, longitudinal cohort studies suggest that dysanapsis may also precede clinical asthma (75).

The first attempt at defining trajectories was from the Tucson study, which described four trajectories: early wheeze (0–3 yr), persistent wheeze (0–6 yr), late-onset wheeze (3–6 yr), and no wheeze (76). These can be criticized, as they were based on data at two time points only (3 and 6 yr), so they were to some extent self-fulfilling, because no more than four trajectories could be discovered by definition. Over the last 15 years, data-driven techniques that group individuals using unbiased approaches have been increasingly used to uncover the temporal patterns of wheezing (recently reviewed in Reference 77). Approaches have included latent class analysis, cluster analysis, and latent trajectory analysis. Most cohorts have reported similar “phenotypes,” including never-wheeze, early-onset remitting (transient) wheeze, late-onset wheeze, and persistent wheeze. An important question is whether different wheeze phenotypes derived by data-driven methods are underpinned by different mechanisms (78). The largest study of this type to date, which used latent class analysis to investigate development of wheezing from birth to adolescence in >15,000 participants in five birth cohorts, recently suggested that genetic associates of different wheeze phenotypes are phenotype-unique (79), highlighting the potential value of using data-driven analyses for deep phenotyping to disaggregate childhood wheeze, with follow-up genetic/mechanistic studies probing underlying mechanisms of discovered classes/clusters. One important finding from data-driven methods is that all clusters/phenotypes of preschool wheezing (including the transient) are associated with impaired lung function trajectories from childhood through early adulthood (80), highlighting the need for research to understand mechanisms of all preschool wheeze phenotypes.

### Family History of Asthma and the Contribution of Genomics

A family history of asthma in parents or grandparents is a consistent risk factor for asthma in childhood—a risk that increases with a higher number of parents and grandparents with asthma (up to 15-fold) (81). This increased risk may be due to shared environmental factors, such as maternal smoking and air pollution, and shared genetic factors, which mainly include prevalent SNPs.

Twin studies have indicated that genetic factors may account for approximately 70% of the variation in asthma susceptibility and approximately 35% of the variation in the age at onset of asthma (82). Large-scale genetic studies that investigate the association of SNPs across the genome with the presence of asthma indicated that the genetic susceptibility to asthma is polygenic and complex, with more than 200 genetic variants that were shown to increase asthma risk (3, 83). Interestingly, these studies show that the genetic susceptibility to childhood-onset asthma is more than three times as strong as adult-onset asthma, which appears to be more strongly driven by environmental factors. In addition, the genetic architectures of childhood- and adult-onset asthma are partly distinct (84, 85). The genetic architecture of asthma beginning in childhood is almost completely overlapping with other childhood-onset allergic diseases, including eczema and rhinitis, indicating that a shared susceptibility to allergy may drive asthma development in childhood (86, 87). Functional interpretation of these studies indicates that childhood-onset asthma is driven by dysregulated genes relating to allergy and epithelial barrier function, whereas adult-onset asthma is more lung centered and driven by environmental factors. Both childhood- and adult-onset asthma share immune-mediated mechanisms driving disease progression (85). Most important examples of genes involved in childhood-onset asthma include the 17q locus that regulates *ORMDL3* and *GSDMB*, *CDHR3* encoding the receptor for RV-C, and genes encoding epithelial cytokines (*IL33*, *TSLP*) and their receptors (*IL1RL1* and *IL7R*) (3).

Next to genetics, epigenetic studies have been performed that relate changes to the genome that affect gene expression, which may be heritable but do not alter the genetic code. These genomic changes include modifications to the DNA (DNA-methylation of a cytosine that is positioned next to a guanine) or to the histones, as well as regulatory RNAs, such as micro-RNAs that regulated gene expression. Environmental factors important to childhood-onset asthma, such as maternal smoking, maternal stress, air pollution, or microbial exposures, may change the epigenome, providing a bridge of environmental factors and genetic regulation of asthma (88, 89). Most studies have investigated DNA-methylation profiles of blood and nasal brushed cells in childhood asthma and revealed replicable patterns of DNA methylation across cohorts, most strongly associated with the influx and activation of inflammatory cells. Similar to genetic studies, blood and nasal analyses point toward shared DNA-methylation patterns of asthma with rhinitis and eczema in childhood (90, 91). Although there is evidence of cord blood DNA-methylation sites being predictive of asthma (92, 93), recent investigations using causal methods suggest that most DNA methylation is the consequence of asthma, suggesting potential future biomarker applications of epigenetics in childhood asthma (88, 94).

**Figure 1.**
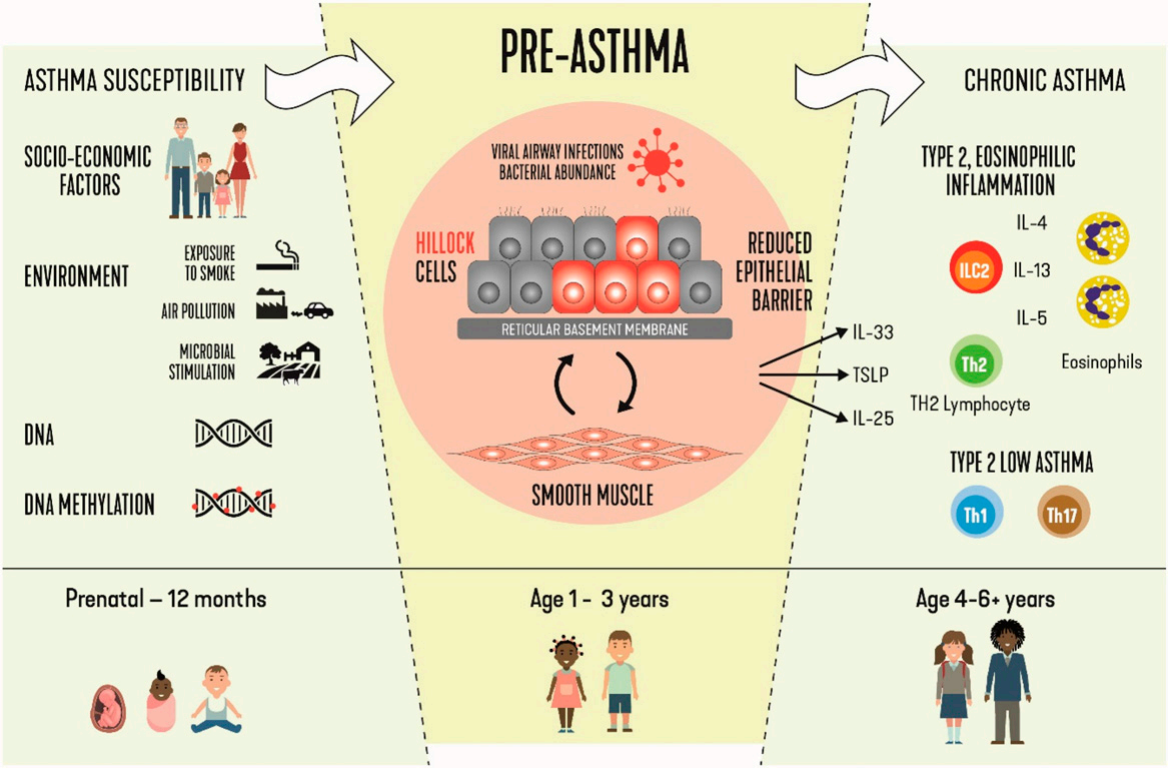
From asthma susceptibility to pre-asthma and chronic asthma (figure by Koppelman).

Transcriptomic studies, which investigate genome-wide gene expression in the airways of children developing asthma, have recently provided a breakthrough in our understanding of the onset of asthma. Transcriptional patterns in airway wall biopsies of 22 symptomatic 1-year-old children were related to asthma at school age. Children who had asthma at school age overexpressed a gene signature characteristic for an airway epithelial differentiation trajectory via hillock cells toward squamous cells (95). Hillock cells represent a recently discovered novel epithelial cell type that expresses markers associated with squamous epithelial differentiation, cellular adhesion, and immunomodulation (96). Interestingly, there was no association with gene signatures of T2 inflammation. Childhood-onset asthma genes are most strongly expressed in cells along the hillock-to-squamous differentiation trajectory, compared with other epithelial cells. These data indicate that in the first years of life, an airway wall response exists in genetically susceptible individuals that is characterized by remodeling, but not (T2) inflammation, which precedes asthma. We therefore propose three phases in asthma development: *1*) susceptibility; *2*) pre-asthma (characterized by epithelial dysfunction, hillock-to-squamous differentiation but not T2 inflammation, and AR); and *3*) persistent asthma (mainly characterized by T2 inflammation, but also T2 low) (95, 97) (Figure 1).

## Risk Factors

### Perinatal and Early-Life Postnatal Exposures

To date there are a number of perinatal and early postnatal risk and protective factors for childhood asthma development that are supported by observational studies, meta-analyses, and a smaller number of randomized controlled trials (RCTs) of interventions to demonstrate causality and proof of concept (1). Below, we summarize the data on asthma risk and protective factors during the *in utero* and early-life period on asthma through childhood and preadolescence (Table 1, including references) (98–126). These effects may not be sustained into adulthood. Maternal and *in utero* risk factors for childhood asthma development include history of maternal asthma, maternal smoking or secondhand smoke exposure, air pollution, maternal stress, gestational weight gain and obesity during pregnancy, birth by cesarean section, and folic acid and antibiotic use during pregnancy. Maternal protective exposures include high intake of vitamin D, fish oil, or both.

**Table 1.** Risk and Protective Factors for Early Childhood Asthma Organized by Maternal/*In Utero* and Infant/Child Factors and Exposures, Summary Estimates, and References

	Risk Factor	Protective Factor	Summary Statistics and Reference
Maternal and *in utero* factors			
Maternal asthma	X		Family history of asthma: incidence rate through the fourth year of life (IRR, 1.94; 95% CI, 1.76–2.16), after which the rates converged (98)
Maternal smoking and secondhand smoke exposure	X		Any maternal smoking: aOR = 1.19; 95% CI, 0.98–1.43 (99)
GWG and maternal obesity	X		Maternal obesity or overweight during pregnancy: aOR, 1.41 and 1.13; 95% CI, 1.26–1.59 compared with normal GWG, very high GWG (aOR, 1.24; 95% CI, 1.04–1.47), moderate high GWG (aOR, 1.12; 95% CI, 1.04–1.21), and very low GWG (aOR, 1.26; 95% CI, 1.08–1.47) (100)
Maternal stress	X		Pooled OR, 1.56; 95% CI, 1.36–1.80 (101)
Maternal antibiotics	X		RR, 1.28; 95% CI, 1.22–1.34 (102)
Folic acid supplementation	X		OR, 1.07; 95% CI, 1.04–1.11 (103)
Birth by cesarean section	X		RR, 1.20; 95% CI, 1.15–1.25 (104)
Vitamin D supplementation during pregnancy		X	Meta-analysis of RCTs of vitamin D supplementation (OR, 0.72; 95% CI, 0.56–0.92). Omega-3 fatty acids did not reach statistical significance (OR, 0.70; 95% CI, 0.45–1.08) (105)
Omega-3 PUFA supplementation during pregnancy		X	Omega-3 fatty acids did not reach statistical significance (OR, 0.70; 95% CI, 0.45–1.08) (105)
Infant and child factors			
Male sex	X		Before puberty onset: aHR, 0.67; 95% CI, 0.61–0.74 for girls (51.3% boys vs. 48.8% girls). After puberty onset, sex difference did not reach statistical significance (aHR, 0.84; 95% CI, 0.64–1.10 (106)
Family history of asthma	X		Family history of asthma (pooled OR, 2.20; *P* < 0.001 (107)
Black ancestry	X		Pooled frequency of clinician-diagnosed asthma in children: highest in black (15%; 95% CI, 3.5–26.5%), then white (10.6%; 95% CI, 4.6–16.7%) and South Asian (7.6%; 95% CI, 3.7–11.4%) (108). U.S. birth cohorts: increased incidence of asthma in Black (HR, 1.47; 95% CI, 1.26–1.73) and Hispanic children (HR, 1.29; 95% CI, 1.09–1.53) compared with White children, with earlier onset of asthma (98, 109)
Preterm birth	X		Risk for asthma in relation to preterm birth (<37 wk of gestation): OR, 1.40; 95% CI, 1.18–1.67 (110)
Lower socioeconomic status	X		Risk for asthma at age 14 yr: OR, 2.30; 95% CI, 1.23–4.31 (111)
Secondhand smoke exposure	X		Overall exposure: OR, 1.24; 95% CI, 1.20–1.28; I^2^ = 86.7%; environmental tobacco smoke: OR, 1.24; 95% CI, 1.19–1.30; I² = 88.2%; postnatal paternal smoking: OR, 1.23; 95% CI, 1.14–1.33; I^2^ = 82.8%; postnatal maternal smoking: OR, 1.17; 95% CI, 1.04–1.29; I^2^ = 91.6% (112)
Low physical activity	X		Risk of new-onset asthma in children with low PA (OR, 1.32; 95% CI, 0.95–1.84 [random effects] and OR, 1.35; 95% CI, 1.13–1.62 [fixed effects]) (113)
Obesity	X		Overweight or obesity: OR, 1.30; 95% CI 1.23–1.39 (114)
Indoor dampness and mold	X		Mold exposure and asthma: aOR, 1.35; 95% CI, 1.20–1.51 (115)
Antibiotics	X		Antibiotic use in early life: OR, 2.18; 95% CI, 1.04–4.60; I^2^ = 76.3% (116)
Acetaminophen	X		Acetaminophen use in early life and childhood: OR, 1.47; 95% CI, 1.36–1.56 and OR, 1.60; 95% CI, 1.48–1.74) (117)
Air pollution	X		The overall random-effects risk estimates (95% CI) were 1.08 (1.03–1.14) per 0.5 × 10^−5^ m^−1^ black carbon; 1.05 (1.02–1.07) per 4 μg/m^3^ nitrogen dioxide; 1.48 (0.89–2.45) per 30 μg/m^3^ nitrogen oxides; 1.03 (1.01–1.05) per 1 μg/m^3^ particulate matter <2.5 μm in diameter; and 1.05 (1.02–1.08) per 2 μg/m^3^ particulate matter <10 μm in diameter (118)
Early-life gastrointestinal or respiratory microbiome	X	X	May be associated with an increased or decreased risk of asthma depending on the diversity and composition of the microbiome (119)
Respiratory viral lower respiratory tract infection (RSV and RV)	X		RSV bronchiolitis compared with healthy control subjects: OR, 7.21; 95% CI, 3.92–13.28. RV-bronchiolitis compared with RSV bronchiolitis on asthma development: OR, 2.72; 95% CI, 1.48–4.99; I^2^ = 65% (6)
Residence in urban and high-poverty areas	X		0–2 yr: did not reach statistical significance (RR, 3.10; 95% CI, 0.44–21.56; I² = 93%); 0–6 yr: RR, 1.21; 95% CI, 1.01–1.46; I² = 95%; 0–18 yr: RR, 1.35; 95% CI, 1.12–1.63; I² = 99% compared with rural areas (120). In U.S. cohort studies, lower socioeconomic status and higher population density associated with a higher HR of asthma incidence (109)
Residence on Amish or European farms with traditional farming practices, and Chinese agricultural farming and poultry		X	Exposure to traditional farming environments in childhood: OR, 0.77; 95% CI, 0.60–0.99; I^2^ = 68% (121). Exposure to agricultural farming (aOR, 0.74; 95% CI, 0.56–0.97) and poultry (aOR, 0.75; 95% CI, 0.59–0.96) (122)
Breastfeeding		X	Longer duration: OR, 0.84; 95% CI, 0.75–0.93; I^2^ = 62.4%. Exclusive: OR, 0.81; 95% CI, 0.72–0.91; I^2^ = 44% (123)
Siblings		X	Number of siblings (:2 vs. 1): 13 studies, pooled RR, 0.97; 95% CI, 0.82–1.14; I^2^ = 89.27%; did not reach statistical significance. Having :1 older sibling: RR, 0.93; 95% CI, 0.88–0.99 protective for :6 yr (124)
Daycare		X	Early daycare attendance (i.e., starting younger than 12 mo) and asthma diagnosis at 3–5 yr of age (OR, 0.66; 95% CI, 0.50–0.87; I^2^ = 0%); asthma diagnosis at 6–18 yr did not reach statistical significance (OR, 0.98; 95% CI, 0.73–2.97; I^2^ = 71.1%) (125)
Dog exposure first year of life		X	Dog exposure during the first year of life was associated with a decreased risk of asthma in school-aged children (OR, 0.87; 95% CI, 0.81–0.93) (126)

*Definition of abbreviations*: aHR = adjusted hazard ratio; aOR = adjusted odds ratio; CI = confidence interval; GWG = gestational weight gain; HR = hazard ratio; IRR = incidence rate ratio; I^2^ = heterogeneity statistics (in meta-analysis); OR = odds ratio; PA = physical activity; PUFA = polyunsaturated fatty acid; RR = relative risk; RCT = randomized controlled trial; RSV = respiratory syncytial virus; RV = rhinovirus.

Recent meta-analyses and systematic reviews were selected as references when available.

Infant and child risk factors include male sex, family history of asthma, Black ancestry, secondhand smoke exposure, low physical activity, obesity, indoor dampness or mold, medications (*see below*), air pollution, the composition of the early-life microbiome, respiratory viral infection with RSV in the first year, RV or other viral wheezing or lower respiratory tract illness, high poverty, and urban residence. Preterm birth is strongly associated with neonatal and later respiratory morbidity, including asthma diagnosis, but it should be noted that subphenotypes exist among preterm children (in one cohort study classified as “asthma-like,” “dysanapsis-like,” and “preterm reference” subgroups using data-driven latent class analysis) (127). Infant and child protective factors include residence on Amish or traditional European farms, Chinese agricultural farming and poultry, duration and exclusivity of breastfeeding, the composition of the microbiome, having one or more older siblings, and daycare in the first 6 months of life. Also, dog exposure during the first year of life has been associated with reduced risk of asthma in school-aged children.

A number of studies have additionally assessed the impact of asthma risk factors on the outcome of asthma phenotypes, suggesting that some risk factors may contribute more strongly to specific asthma phenotypes than others. This is important in estimating the individual contribution of potentially modifiable risk factors and delineating the mechanisms through which risk factors drive asthma development.

Importantly, early-life adverse exposures (tobacco smoking in particular) may lead to long-term impairment in lung health and lung function, even having transgenerational effects on the maternal and/or paternal side (potentially through epigenetic mechanisms) (128, 129). *In utero* or early-life exposures, such as nicotine or LRTIs, lead to impairment in lung health and pulmonary function (130), setting a trajectory that may track for life and includes development of asthma, chronic obstructive pulmonary disease (COPD), and premature mortality. The mechanisms of risk and protection of environmental factors are likely related to the impact of environmental exposures on the immature adaptive immune system, airway structure or function, and the gut and airway microbiome in early life.

Last, population attributable fraction (PAF) is important as a public health target, identifying risk factors with a high PAF, high exposure prevalence, and, thus, most likely to have a large modifiable impact. A prior study used 32 meta-analyses to calculate PAF and exposure prevalence during pregnancy and early-life child stages to illustrate the impact of each modifiable risk or protective factor on asthma development (131). The early-life risk factors with the greatest PAF included acute viral respiratory tract infections, antibiotic use, inadequate physical activity, prenatal stress, secondhand smoke exposure, and allergic sensitization. Exposures with the greatest protective PAF included breastfeeding and sufficient maternal vitamin D levels.

### Lifestyle Factors, Environmental Exposures, and Climate Change

Among the lifestyle factors, smoking status, obesity, low physical activity, and unhealthy dietary patterns are well-established risk factors for asthma in both children/adolescents and adults. Antioxidants and vitamin D, which play an important role in immune regulation, have been linked to both asthma and obesity. For example, decreased oral intake and increased oxidative stress among obese patients with asthma result in lower serum concentration of vitamin E, vitamin C, and selenium (132). Obesity has also been associated with increased adipose tissue sequestration and decreased vitamin D bioavailability (133). Although lower levels of these specific nutrients have been associated with asthma, clinical trials of supplementations with these nutrients targeting older children and adults did not result in clinical improvement (134–136). This highlights the importance of prenatal and early-life diet in the development of obesity and asthma inception. In fact, maternal vitamin D supplementation during pregnancy—a critical period when the immune system and the lungs are developing—was associated with a lower risk of wheezing in the offspring during childhood (135, 136).

Regarding smoking, e-cigarettes, introduced in the market in early 2000s, have been shown to increase the risk of asthma in adolescents and in adults in cross-sectional studies (137). Although most studies conducted so far focused on investigating the influence of a single lifestyle factor, some recent studies based on large population-based samples reinforced the critical role of lifestyle factors in asthma by jointly evaluating the impact of multiple lifestyle factors (138, 139). Considering the combined effects of smoking, body mass index or body silhouette, physical activity, and diet, these two studies showed that adults with a poor lifestyle had a significantly higher risk of incident-onset asthma over 10 years (138) and increased asthma symptom score and risk for uncontrolled asthma (139). Interestingly, the increased risk of asthma associated with a poor lifestyle was observed both in low, intermediate, and high genetic risk subgroups defined by a polygenic risk score (138), and the effect size of lifestyle factors was higher than that of genetic risk and socioeconomic, external environment, and early-life environment profiles (139).

Although asthma often begins as an inappropriate response to viral infections or allergic sensitization, low socioeconomic status and medication exposure in early life have also been associated with the inception of asthma (140). Children who live in low-socioeconomic families are twice as likely to develop asthma by 14 years of age, whereas children who live in high-socioeconomic families are less likely (60%) to develop asthma (115). Furthermore, lower socioeconomic status has been associated with increased healthcare use, but not asthma mortality (141). This raises important questions regarding exposures to low-income environments from birth, which may underlie the development of asthma. In particular, poor housing conditions, which disproportionately affect low-income communities, may influence asthma incidence and progression through exposure to indoor allergens (pests, dust mites, molds). Several interventional studies have provided initial evidence of the efficiency of home repairs on asthma morbidity, thus calling for large research programs to guide public health policies to improve housing conditions to achieve better health equity (142).

In addition, because socioeconomic factors often correlate with race and ethnicity, disentangling the independent contribution of each factor in the risk of asthma is challenging. For example, similar to childhood asthma, obesity unequally affects ethnic minorities. In the United States, 26.2% of Hispanic children, 24.8 of non-Hispanic Black children, and 16.6% of non-Hispanic White children were obese in 2020 (143). The existing literature on this overall topic remains inconclusive (114, 144) and points to the need for further studies to investigate to what extent socioeconomic factors could mediate the association between race/ethnicity and asthma.

In line with results observed in children (145), evidence for chronic effects of long-term exposure to air pollutants (particulate matter < 2.5 μm in diameter, NO_2_, black carbon) in asthma incidence and prevalence in adults was reinforced by recent studies on large population-based samples (146, 147). Many studies have mainly evaluated traffic-related air pollutants (e.g., Reference 145), although exposure from power plants or natural sources (wildfires, sandstorms—mainly particles) may also contribute significantly. Indoor pollutants typically derive from cookstoves and combustion activities (open fires, candles, smoking, etc.). The extent to which air pollution and physical activity interact in asthma incidence remains poorly addressed. Although a large cohort from Denmark showed a beneficial effect of physical activity in reducing asthma incidence regardless of outdoor concentration of air pollution (NO_2_) (148), a smaller study using wearable sensors found that the beneficial effect of physical activity decreased with higher air pollution levels (black carbon) (149). Although a synergistic effect between outdoor air pollution and allergen exposures on asthma is supported by experimental studies, results from epidemiological studies are inconsistent (150).

Accumulated epidemiological evidence indicates that climate change affects asthma directly by intensifying extreme climate events, such as heat waves, wildfires, and thunderstorms, and indirectly by its impact on major asthma risk factors, such as aeroallergens (e.g., increasing pollen concentration and prolonged pollen seasons, increased mold proliferation) and air pollution (151–153). A recent large-scale study found that risk of asthma hospitalization in England between 2002 and 2019 increased by 1.11% (95% CI, 0.88–1.34%) for every 1°C increase in the daily mean ambient summer temperature (154). This risk was the highest for males aged 16–64 years and has decreased overt time in the study period, which needs further investigation but might be explained by adaptative mechanisms or changes in heat exposure over time.

More recently, concern has been raised about the impact of exposure to ubiquitous environmental chemicals on asthma. Pesticides are suspected to be involved in asthma in children (155) and adults (156). Early-life exposure to endocrine-disrupting chemicals (phenols, phthalates, and poly- and perfluoroalkyl substances) has gained attention because of their immunomodulatory effects, but the epidemiological evidence is still limited (157).

The exposome concept proposes a comprehensive approach to characterize the effects of the environment on complex multifactorial diseases. Because some environmental exposures are interconnected (e.g., climate change and air pollution that both originate from fossil fuel combustion [5]), and some environmental factors might interact, the exposome approach should provide a step forward for a better understanding of the development and progression of asthma, which in turn can lead to multifaceted preventive strategies (158).

### Medications

Several medication exposures in early life have been associated with asthma inception. Observational studies have noted an increased risk for asthma of 30% or more in children and adolescents who used acetaminophen, a commonly used analgesic and antipyretic, in the prior year or during the first year of life (122, 159–161). Likewise, prenatal use of acetaminophen was associated with 20–50% increased risk of asthma and wheezing in their offspring (122). More recently, the presence in umbilical cord blood of acetaminophen metabolites has, on multivariate analysis, been associated with an increase in the odds of children having asthma at 6-year follow-up (162). In contrast, ibuprofen has not been consistently associated with asthma development (122, 163). In an adult population 16–40 years of age, asthma risk correlated positively with the frequency of acetaminophen use (*P* trend = 0.0002), which was highest among weekly (OR, 1.79; 95% CI, 1.21–2.65), and daily users (OR, 2.38; 95% CI, 1.22–4.64) (164). Although the exact mechanism is not well known, it has been postulated that acetaminophen may play a role in asthma pathogenesis by reducing the levels of the endogenous antioxidant glutathione in lung tissue. Large RCTs are needed to provide safety and efficacy data for acetaminophen use in early life, especially in premature infants.

Antibiotic use has been proposed to deplete protective bacteria in the microbiome, which may alter the normal microbial–mucosal interface and promote the development of asthma (165). In a Korean cohort of 789 children, prenatal exposure to antibiotics was associated with a fivefold increase in the risk of physician-diagnosed asthma at 7 years (166). Postnatal antibiotic exposure has also been associated with asthma in two large cohorts from Sweden and New England (167, 168). However, this association has not always been consistent. In a separate Swedish birth cohort of 4,089 infants, early-life antibiotics were association with higher risk for asthma at 4 years, but not at 8 years (169). Although antibiotic therapy can be lifesaving in the perinatal and neonatal period for pathogenic bacteria, the inappropriate use of broad-spectrum antibiotics can potentially alter the microbiome and increase the risk of asthma in the long term.

## Asthma Across the Life Course

### Epidemiology of Asthma during the Life Course

**Figure 2.**
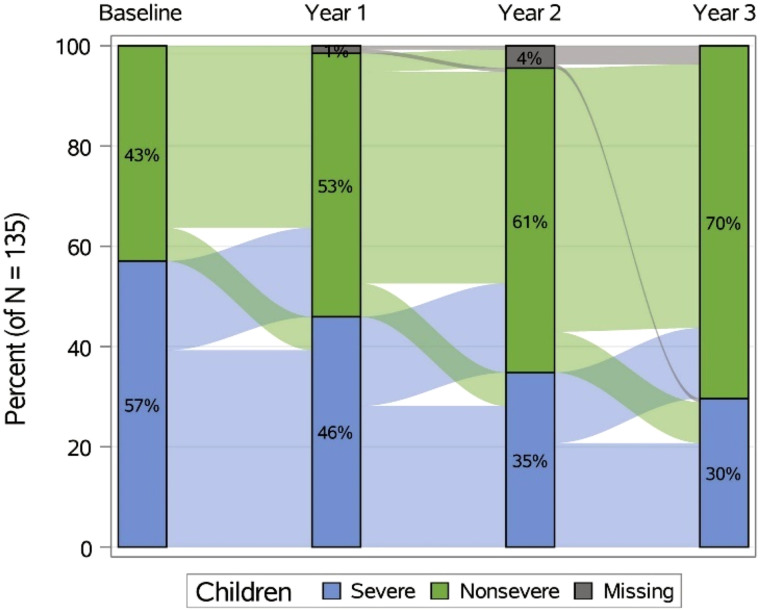
Year-to-year change in asthma severity in the SARP III (Severe Asthma Research Program III) pediatric cohort. Sankey plot shows year-to-year change in asthma severity in the SARP III pediatric cohort. Stacked bars show percentages of participants with severe and nonsevere asthma at each yearly visit, whereas connecting regions show the proportion who changed categories from one year to the next. Reprinted by permission from Reference 209.

Asthma is the most common chronic disease in children and adolescents, with a rising prevalence in both urban and rural areas, albeit with large regional differences. Globally, questionnaire-based studies, particularly phase 1–3 of ISAAC (International Study of Asthma and Allergies in Childhood) and most recently GAN (Global Asthma Network) with data collected 2016–2020, showed that the prevalence of asthma varies widely by income level, geographic region, country, within countries, and even within cities among children and adolescents, as measured in two specific age groups: 6–7 years and 13–14 years (Figure 2). Although asthma was considered to be less prevalent in LMIC settings, data have shown a rising prevalence in several LMICs, with more severe disease than that in high-income settings (170–173). For example, in African children, asthma prevalence is higher than the global prevalence (172), with almost half of children who reported asthma in ISAAC 3 having severe symptoms. More than 30% of those with severe asthma symptoms had never had a diagnosis (170, 172, 173). In adults, wide geographic variation of asthma prevalence has also been reported. In the ECRHS (European Community Respiratory Health Survey), conducted in 48 centers in the early 1990s, the asthma prevalence in adults ranged from 2% to 12% (174). Recent results from the GAN study from 2016 to 2020 similarly showed strong variation of asthma diagnosis prevalence in centers between countries (from <1% from centers in Kosovo and India to 10–19% from centers in Spain, Kingdom of Saudi Arabia, Brazil, Costa Rica, New Zealand, and Honduras) and within countries (for example, from 1.4% to 6.9% from centers across Mexico), as well as by gross national income level (175). Of note, the prevalence of asthma phenotypes varies across the world, for example, data from the WASP (World Asthma Phenotypes Study) show that many patients in Africa have noneosinophilic disease, with a particularly high prevalence of airway neutrophilia in Uganda, both in patients with asthma and in the normal population (126). These highlight the importance of identifying asthma phenotypes and developing interventions that address noneosinophilic asthma globally.

### Adolescent- and Adult-Onset Asthma

Longitudinal trajectory analyses from birth cohorts show that adolescent-onset asthma is frequent (6.4% of the study population) and that uncontrolled asthma was equally prevalent in the adolescent-onset and childhood persistent asthma group (176). However, the persistent asthma trajectory group showed more signs of T2 inflammation than the adolescent-onset trajectory group, suggesting different underlying endotypes. Predisposing factors for adolescent-onset asthma are atopy, rhinitis, and bronchial hyperresponsiveness by school age, suggesting that airway symptoms and disease processes likely start long before the diagnosis is made (177). Also, a majority of patients with asthma onset in early adulthood (by age 22 yr) had wheezing episodes before the age of 6 years (178).

Most people with asthma receive their diagnosis in adulthood (179). Based on questionnaire data from a large Finish population, the incidence of asthma diagnosis was calculated in 10-year age groups and peaked in young boys (0–9 yr) and middle-aged women (40–49 yr) (179). Adult-onset asthma can be split into early-onset and late-onset, (late-onset asthma normally refers to asthma occurring after the age of 40 years [180]) and may differ from childhood-onset asthma (often called longstanding asthma) in terms of pathophysiology and response to treatment (181). Separating adult-onset asthma from COPD can be challenging, and studies are hampered by methodological difficulties, especially when interpreting lung function changes with age. Aging is associated with several physiological lung changes, including a decrease in elastic recoil, greater chest wall rigidity, and reduced respiratory muscle strength, and as FEV_1_ declines with age, starting around 25–30 years (182). One study found that early-onset adult asthma was more strongly associated with atopy and family history of asthma than late-onset adult asthma, which was more strongly associated with female sex, current smoking, and lower socioeconomic class (183). Although a longer duration of asthma is associated with worse lung function, similar symptom burden occurs with asthma of shorter duration (180, 183). Cluster analysis has also revealed a population of female, obese, nonatopic late-onset asthma, with less eosinophilic airway inflammation (184). Asthma remission is recognized (1, 185), and the likelihood of remission changes over the life course, with a 60% clinical remission of asthma (i.e., no symptoms and no use of asthma medication) in people who develop the disease before the age of 10 years, but only 5–15% for adult-onset asthma (1). Critically, whether clinical remission equates to pathophysiological remission remains unknown, and a lack of resolution of childhood airway pathology may explain some of the incidence of “adult-onset” asthma.

Occupational asthma is a well-recognized adult-onset phenotype, typically linked to exposure to toxic inhalants in the workplace (186), but this type of asthma is outside the scope of this review.

### Exercise-induced Bronchoconstriction and Asthma

Exercise-induced bronchoconstriction (EIB) complaints are common in children and adolescents. EIB occurs in up to 40–90% of people with asthma (187), with a higher prevalence in elite athletes (188). Up 20% of people with EIB have no underlying diagnosis of asthma (188, 189). EIB is believed to be caused by inflammatory, neuronal, and vascular changes resulting in contraction of the bronchial smooth muscle and epithelial damage and results from water loss, thermal changes, and irritant exposure induced by increased minute ventilation and repeated warming/humidification of inspired air during exercise. Unlike non–exercise-induced asthma, EIB symptoms usually resolve spontaneously within 30–90 minutes of exercise and induce a refractory period of 1–3 hours, where continued exercise does not produce bronchoconstriction. Sports involving exposure to cold air, chlorinated water, or with higher respiratory loads (e.g., swimming, cross-country skiing, and triathlon) are associated with increased risk.

## Prevention of Asthma Onset and Progression of Established Disease

### Primary Prevention of Asthma

Asthma rates are highly variable across the world and even within countries, pointing to environment and lifestyle as contributing factors. Epidemiologic studies have identified risk and protective factors, and untargeted public health initiatives have been implemented, whereas interventional RCTs have targeted pregnant women and children with the goal of the prevention of childhood asthma. Untargeted prevention strategies, such as smoking bans and laws that regulate air emissions, such as the U.S. Clean Air Act, result in a reduction in the overall exposure of pregnant women and children to asthma risk factors, while also contributing to other positive health outcomes. However, although there are data on the impact of such programs on asthma exacerbations, data are sparser on their impact on asthma inception (190–192). These public health interventions largely target decreasing exposure to established risk factors for asthma inception, whereas RCTs largely target introduction of protective factors.

RCTs include trials of vitamin D and n-3 long-chain PUFA supplementation to pregnant women and infant RSV immunoprophylaxis, which may protect against recurrent wheeze, yet, results on school-aged asthma were less clear (11, 110, 193). Although there was hope that using inhaled corticosteroids in the at-risk preschool population could prevent asthma, large prospective studies demonstrated that inhaled corticosteroids do not prevent childhood asthma development (61–63, 194). Oral application of bacterial lysates is ongoing (NCT02148796), but the outcome is a pre-asthma phenotype of first wheezing lower respiratory tract illness and not asthma, and many interventions have demonstrated an effect on wheeze, but not asthma.

We know less about the prevention of adult-onset asthma, and there are no primary prevention studies targeting prevention of adult asthma of which we are aware (195). For adult-onset asthma, primary prevention is focused mainly on smoking cessation, preventing exposure to secondhand smoke, and control of workplace exposures that contribute to occupational asthma. There are studies in adolescents using bacterial lysate to steer host immunity away from T2 response, with nutritional interventions and with immunotherapy against respiratory viruses, but longer-term follow-up will be required to know whether these interventions prevent asthma in adolescence and adulthood (196).

Given the wealth of information on asthma risk factors, why have we not made progress in primary asthma prevention? Some risk factors associated with asthma inception are probably best tackled at a public health or population level, such as obesity prevention, pollution, violence, and physical activity, as they are likely to have many positive health benefits. Targeted immunologic therapies that may modify the immature and developing immune system, airway, and microbiome would need to be approved for infants or young children to intervene before early-childhood asthma development, and new biologic therapies are often not studied or approved in very young children. Last, not all risk and protective factors identified in observational studies may be causal in asthma development. Given the strong environmental contribution to asthma development, and the significant societal and individual burden, primary prevention strategies at the population and individual level should be a priority.

### Transition from Childhood to Adulthood

It is well established that around half of children with asthma <10 years of age may grow out of their disease eventually, as indicated by absence of asthma symptoms and no use of asthma medication. However, asthma may return later in life (1). Prognostic scores have been evaluated to better predict which children will have persistent asthma through to adulthood. Typically, variables like parental asthma (heredity), childhood wheezing patterns, allergic comorbidities (e.g., eczema, hay fever), sensitization (skin prick test or specific IgE measures), and recurrent airway infections are included in the models with overall performance, assessed by area under the receiver operating curve ranging between 0.66 and 0.87 (197). One example is the validated Pediatric Asthma Risk Score, which was shown to provide robust estimates of asthma risk in children up to age 10 years from a wide range of ethnicities and backgrounds (198). Other models have been developed to predict long-term asthma risk from childhood to adulthood (199).

The combination of normal FEV_1_/FVC ratio, no airway responsiveness, and low blood eosinophil count is, on the other hand, linked to >80% probability of remission by adulthood (200). Eosinophilia has been associated with persistence of asthma (201), in addition to allergic comorbidity and IgE sensitization (in particular, poly-sensitization to several allergens) and should be viewed as a strong prognostic marker for disease persistence together with elevated fractional exhaled nitric oxide (202). Other risk factors associated with persistence of asthma from childhood to adulthood include family history/genetics, asthma severity, repeated airway infections, air pollution and tobacco smoke exposure, as well as obesity (203, 204).

In the last few years, there has been a focus on finding new biomarkers that might aid asthma diagnostics and, importantly, provide informative predictive value about long-term prognosis. One such biomarker is CC16 (club cell 16), which is an antiinflammatory protein highly expressed in the airways and previously linked with lung function deficits in both children and adults (205). Recently published results from three longitudinal cohort studies show that children with asthma in the lowest CC16 tertile had a nearly fourfold increased risk for having frequent asthma symptoms persisting into adult life (participants followed up to age 36 yr) (206). Children with persistent asthma, in particular those with recurrent exacerbations, are at increased risk for fixed airflow obstruction and possibly COPD later in adulthood (207, 208).

### The Evolution of Severe Asthma across the Life Course

Severe asthma is more common in boys than girls (209). In adulthood, however, this pattern is reversed: roughly two-thirds of severe asthma in adulthood is in women. In children with severe asthma followed longitudinally during the transition from childhood to adulthood, severe asthma resolves in two-thirds of patients, and both boys and girls improve equally (209).

Several variables have been extensively considered to help understand this improvement in severe asthma among adolescents (209). Clearly, one is growth of airway caliber, and worsening dysanapsis could contribute to relative worsening in women (210). Behavioral and environmental characteristics expected during adolescence (e.g., smoking, drugs, and poor adherence to therapy) would likely have an effect opposite of that observed. One potential factor underlying decreased severity during adolescence has received recent attention: increasing androgen levels in boys and girls during adolescence may benefit lung function (211). Better lung function and better asthma symptom scores are associated with higher circulating androgen levels in asthma (211, 212). This association could reflect treatment with corticosteroids, which can decrease circulating DHEAS (dehydroepiandrosterone sulfate) levels. However, higher testosterone levels, not so much affected by ICS (inhaled corticosteroids), are also associated with better lung function (212). Furthermore, an androgen metabolic gene variant that increases tissue androgens—and increases mortality risk in prostate cancer—is associated with improved outcomes in asthma (213). Moreover, in mice (214) and humans (215), androgen receptor deficiency decreases asthma risk, and children and adults with decreased airway androgen receptor expression have more severe asthma, lower lung function, more inflammation, and worse symptom scores (212, 216).

In terms of asthma severity in adult-onset asthma, one study described an independent association between increased blood neutrophil and sputum eosinophil counts in severe adult-onset disease when compared with mild-to-moderate persistent adult-onset asthma (217). Most of the patients with severe asthma were nonatopic and had elevated sputum eosinophil counts.

### Biologics as Disease-Modifying Drugs

One central question is whether (early) use of biologicals may alter the natural history of disease. This has been sparked by the observation that a minority of patients with severe asthma, sometimes called superresponders, respond greatly to biologicals (185, 218). This has been called clinical asthma remission, which is commonly defined as a high level of disease control, with the absence of signs and symptoms for >12 months and optimization of lung function, while still on asthma treatment (185). Some authors have suggested reducing monoclonal doses in patients with prolonged control (219), following evidence that cessation of omalizumab (anti-IgE) in children, after a prolonged period of treatment, was associated with a permanent reduction in exacerbation rates (220). This is especially relevant in the pediatric population, because novel anti-epithelial cytokines TSLP and IL33 represent relevant mechanism in childhood-onset disease (3), and it is possible that intervening in these early mechanisms may prevent chronic, severe inflammatory disease. One major concern in the treatment of asthma, especially childhood-onset disease, is the development of AR. To date, no therapies have shown a reduction in AR (although studies are hard to design because of a lack of noninvasive biomarkers and the variability of disease activity over time); however, there are some early data suggesting that targeted monoclonal antibodies may change airway biology and reduce AR. For example, there was no decline in lung function with dupilumab (anti-IL4/IL13) treatment (adults) as measured by post-bronchodilator FEV_1 _slope from Week 4 to Week 52; in contrast, the placebo groups experienced an estimated 40 ml/yr decline (221), suggesting a possible reduction in AR. However, changes in AR have not been assessed. The positive effects of dupilumab in COPD have drawn more attention to the role of targeted biologics in persistent airflow obstruction (222), so further AR studies are likely to follow.

## Conclusions

From a clinician’s point of view, asthma symptoms can debut at any age and, accordingly, symptom history and adequate lung function tests (typically pre- and post-bronchodilator spirometry) should be undertaken to confirm or refute diagnosis in patients with asthma-like symptoms. However, most patients with asthma, regardless of their age of onset, seem to have had some form of airway symptoms during childhood. Asthma inception and related pathophysiologic processes are therefore very likely to occur in the first years of life, further evidenced by recent transcriptomic and biomarker research, which shows a pre-asthmatic disease state characterized by aberrant epithelial differentiation pattern (Figure 1). These insights have profound consequences for any asthma prevention program. Herein, we have highlighted the critical pathway of early-life viral and bacterial infections in genetically vulnerable individuals to induce an abnormal airway response as key events in asthma. As such, studies evaluating whether infant RSV immunoprophylaxis (223) and/or maternal pregnancy vaccination (224) (and other immunomodulatory prophylactics) may reduce childhood wheezing and asthma incidence are much anticipated. In Table 2, we summarize asthma research priorities going forward.

**Table 2.** Research Priorities in the *American Journal of Respiratory and Critical Care Medicine* Asthma Inception Review

Asthma inception 1. Preconception and perinatal determinants of asthma inception—mechanisms and identification of pathways to target for primary prevention 2. Understanding the mechanistic pathways driving the inception of asthma, including aberrant airway and immune development in preschoolers who will develop school-age asthma 3. Identifying treatment targets for inception and progression of asthma, derived from omics applications, before the onset of airway inflammation 4. Improved understanding of the role of neural control of airway biology and the role of the lung–brain axis in asthma development 5. Determinants of sex differences and endocrinologic mediators driving asthma inception across the life span 6. Gather health systems data to determine whether RSV active or passive vaccination reduces the risk of childhood asthma 7. Need to understand the contribution of both genetic and environmental risk factors to specific asthma phenotypes, as well as establish their causal association 8. Need for primary prevention studies and studies of the impact of public health initiatives on childhood asthma inception Asthma prognosis, severity, and disease control 9. Determinants and prevention of exacerbation-prone asthma 10. Mechanisms linking eosinophilia to viral exacerbations in patients with asthma 11. Role of airway biochemistry in developing new breath biomarkers and new treatments for virally induced asthma exacerbations and other acute asthma events 12. Use of AI and physiological variables to predict and provide insight into timing and management of asthma exacerbations 13. Asthma endocrinology and the determinants of sex differences in asthma severity and treatment responses across the life span Diagnosis and pharmacologic and nonpharmacologic management (children and adults) 14. Realistic, patient-friendly home monitoring in asthma 15. Methods to improve adherence to asthma treatment plans and the effectiveness of asthma medication delivery devices 16. Optimize management and prevention of treatment-related side effects of ICS/OCS: cataracts, diabetes, osteoporosis, etc. 17. Understanding the mechanisms of how the microbiome modulates risk and developing mechanism-based, targeted treatments 18. Disease-modifying asthma treatments and biomarkers to monitor such effects 19. Applying biomarkers of lung structure (including imaging) and AHR/smooth muscle function to monitor disease development and progression 20. Better approaches to manage patients at the intersection of chronic suppurative lung disease and asthma (particularly including children), including persistent bacterial bronchitis, cystic fibrosis, and primary ciliary dyskinesia 21. Effect of biologics in decreasing the severity of asthma, including the difference between complete response to treatment and true remission of asthma 22. Improve our understanding, diagnosis, and treatment of nonatopic or T2-low asthma in children and asthma treatment responses in specific childhood asthma phenotypes (e.g., obesity asthma) Public policy priorities 23. Effective and enforced legislation to keep cigarettes and vape products out of the hands of children and young people (as well as adults) and targeted public health campaigns 24. Effective and enforced legislation to reduce air pollution levels, especially in residential areas 25. Resourced intervention programs for obesity; this will include a “sugar tax” and also a “calorie tax” 26. Establishment of “safe zones” around schools, where there will be no fast-food or nicotine outlets 27. Investigation of the effectiveness of use of spirometry check-ups at school age to identify a group at high risk of respiratory morbidities (which would also have the spin-off benefit of identifying risk for all-cause morbidities), combined with investigations on effective interventions once low lung function is detected 28. Ensure that children who have a significant respiratory infection in the first 2 years of life are seen as being in a high-risk group and monitored accordingly 29. Ensure all patients across the globe have access to basic asthma medications and establish programs whereby biologics can be made available in low- and middle-income settings at an affordable price 30. Mandate a detailed review by an asthma-trained health-care professional after an asthma attack 31. Mandate and promote full immunization, including annual influenza vaccination, of all infants and children (and in particular those with moderate-to-severe asthma) unless there is a proven medical contraindication 32. Need to identify and incorporate consumer (persons with asthma, parents of children with asthma)-driven research priorities

*Definition of abbreviations*: AHR = airway hyperresponsiveness; AI = artificial intelligence; ICS = inhaled corticosteroids; OCS = oral corticosteroids; T2 = type 2.

Although it is known that some children, both during preschool years and school age, may have clinical symptom remission, whether this equates to the disappearance of underlying pathology, especially the structural airway changes, is unknown, and at present there are no interventions that can modify this long-term impact on lung health. Yet, we know that several factors influence lung health in the long run, and we should acknowledge the very clear negative health effects from tobacco smoke and nicotine products (vaping included), air pollution and other chemical exposures, social deprivation, LRTI, poor diet, and obesity (208, 225). For children at high risk of chronic (severe) asthma, a multifaceted approach to reduce exacerbations and deterioration risk is enforced. Available (and coming) biologics hold some promise to have disease-modifying effects in asthma, although additional research is needed before firm conclusions can be drawn. In addition, current mechanistic and biomarker data highlight the heterogeneity of asthma and the potential dangers of treating all patients using the same treatments. Therefore, a precision-medicine approach must be implemented to optimize treatment and tailor follow-up (1).
